# Abundance of Cysteine Endopeptidase Dionain in Digestive Fluid of Venus Flytrap (*Dionaea muscipula* Ellis) Is Regulated by Different Stimuli from Prey through Jasmonates

**DOI:** 10.1371/journal.pone.0104424

**Published:** 2014-08-25

**Authors:** Michaela Libiaková, Kristýna Floková, Ondřej Novák, L'udmila Slováková, Andrej Pavlovič

**Affiliations:** 1 Department of Plant Physiology, Faculty of Natural Sciences, Comenius University in Bratislava, Bratislava, Slovakia; 2 Laboratory of Growth Regulators, Centre of the Region Haná for Biotechnological and Agricultural Research, Institute of Experimental Botany ASCR and Palacký University, Olomouc, Czech Republic; 3 Department of Biophysics, Centre of the Region Haná for Biotechnological and Agricultural Research, Palacký University, Olomouc, Czech Republic; Pennsylvania State University, United States of America

## Abstract

The trap of the carnivorous plant Venus flytrap (*Dionaea muscipula*) catches prey by very rapid closure of its modified leaves. After the rapid closure secures the prey, repeated mechanical stimulation of trigger hairs by struggling prey and the generation of action potentials (APs) result in secretion of digestive fluid. Once the prey's movement stops, the secretion is maintained by chemical stimuli released from digested prey. We investigated the effect of mechanical and chemical stimulation (NH_4_Cl, KH_2_PO_4_, further N(Cl) and P(K) stimulation) on enzyme activities in digestive fluid. Activities of β-D-glucosidases and N-acetyl-β-D-glucosaminidases were not detected. Acid phosphatase activity was higher in N(Cl) stimulated traps while proteolytic activity was higher in both chemically induced traps in comparison to mechanical stimulation. This is in accordance with higher abundance of recently described enzyme cysteine endopeptidase dionain in digestive fluid of chemically induced traps. Mechanical stimulation induced high levels of *cis*-12-oxophytodienoic acid (*cis*-OPDA) but jasmonic acid (JA) and its isoleucine conjugate (JA-Ile) accumulated to higher level after chemical stimulation. The concentration of indole-3-acetic acid (IAA), salicylic acid (SA) and abscisic acid (ABA) did not change significantly. The external application of JA bypassed the mechanical and chemical stimulation and induced a high abundance of dionain and proteolytic activity in digestive fluid. These results document the role of jasmonates in regulation of proteolytic activity in response to different stimuli from captured prey. The double trigger mechanism in protein digestion is proposed.

## Introduction

Carnivorous plants have attracted the attention of scientists for centuries by the ability of these plants to catch and then digest animal prey by enzymes similar to those in the digestive system of vertebrate animals. Although the composition of digestive fluid in some carnivorous genera is now well recognized, the information about regulation of their activity by stimuli from prey are scarce. In this paper we focus on the regulation of proteolytic activity in the carnivorous plant Venus flytrap (*Dionaea muscipula* Ellis), which captures insect by rapid movement of its bilobed modified leaves called a trap. Charles Darwin was fascinated by this plant and he denoted it as the “most wonderful plant in the world” [1]. In response to Darwin‘s request, Burdon-Sanderson discovered electrical activity in this plant [2]. The trap closes in response to a mechanical stimulus delivered to the trigger hairs, protruding from trap epidermis. This initiates a receptor and then an action potential (AP). At least two APs within approximately 20 seconds are necessary for trap closure at room temperature [3], [4], [5]. An external electrical stimulus can also close the trap without mechanical stimulation of the trigger hair, and repeated application of a lower voltage demonstrates the existence of electrical memory in *Dionaea* [6], [7], [8], [9]. The exact mechanism of rapid trap closure remained obscure for many years. The driving force for closure is most probably elastic energy accumulated due to hydrostatic pressure differences between the upper and lower layers of the lobes [10], [11], [12]. Another plausible explanation is the expansion of the cell wall through acid growth [13]. Regardless the mechanism, after rapid closure secures the insect prey, the struggling of the entrapped prey in the closed trap results in generation of further APs which cease to occur when the prey stops moving. Over 100 APs were recorded in the trap with prey in the first 2 hours. This results in further closure and tightening the trap to a tightly appressed state called the narrowed phase. The force increases in the narrowed phase from zero to 450 mN with maximal constriction pressure created by the trap lobes reaching to 9 kPa [14]. Secretion of digestive fluid also appears to be triggered by APs. The narrowed phase and secretion of digestive fluid are also induced by chemical stimulation; Na^+^ and NH_4_^+^ stimulate narrowing at very low concentration; both ions are present in insect haemolymph. As the body of the insect breaks down through the action of digestive enzymes, more chemical stimuli are released into the trap interior which maintains secretion of fluid and trap narrowing. When the prey dies, no APs are generated [15], [16], [17]. Ueda *et al*. have isolated trap-closing chemical factors from jasmonates, which induce trap closing without mechanical stimuli [18]. It has been proposed that the ‘touch’ hormone 12-oxophytodienoic acid (OPDA), which is a precursor of jasmonic acid (JA), induces the secretion of digestive fluid [19].

The digestive fluid released from Venus flytrap in response to different stimuli has proteinase, peptidase, peroxidase, esterase, glycosidase, chitinase and anhydrase activities [20], [21]. The action of the proteolytic enzymes has been researched by several laboratories. Robins and Juniper (1980) and Takahashi *et al*. (2009), for example, noted that proteinase activity is not completely inhibited by pepstatin, a powerful inhibitor of pepsin in vertebrate animals and aspartic proteases nepenthesin in the carnivorous pitcher plant *Nepenthes*, suggesting the involvement of other enzymes in proteolysis [21], [22], [23]. Takahashi *et al.* (2011) published the partial aminoacid sequence of cysteine endopeptidase belonging to the papain family from digestive fluid of *Dionaea* and proposed the name dionain [24]. Recently, Schulze *et al*. (2012) provided aminoacid sequences of four other cysteine endopeptidases in digestive fluid of Venus flytrap designed as dionain 1-4 and two less abundant aspartic proteases dionaeasin-1 and dionaeasin-2 [25]. The cysteine endopeptidase droserain in carnivorous sundew *Drosera indica* has also been recently proposed [26].

Although the enzymatic properties and composition of digestive fluid in Venus flytrap is now well recognized [20], [21], [25], there is little information on how the enzyme activity is regulated by different stimuli from prey. The aim of our study was to investigate the digestive capabilities of fluid released in response to mechanical and chemical stimuli, with particular interest in cysteine endopeptidase dionain. The role of jasmonates in induction of enzyme secretion was also investigated.

## Materials and Methods

### Plant material and experimental setup

Fifty Venus flytrap (*Dionaea muscipula* Ellis) plants were cultivated in greenhouses as part of a collection of carnivorous plants at the Department of Plant Physiology, Comenius University in Bratislava, Slovakia, and Department of Biophysics in Olomouc, Czech Republic in well-drained peat moss in plastic pots (10 x 10 x 10 cm) placed in container filled with distilled water to a depth 1–3 cm. Daily temperatures fluctuated between 15–35 °C, relative air humidity 50–100 % and maximum daily irradiance reached max. 1500 µmol m^-2^ s^-1^ PAR (photosynthetically active radiation). For experiments the plants were replaced to aquarium with 100% relative air humidity. Secretion of digestive fluid was stimulated by mechanical and chemical stimuli. For mechanical stimulation, the trigger hairs in closed trap were stimulated gently by moving the rounded pipette tip (melted by heat and then hardened at room temperature to exclude wound response) every 10–15 min for at least 3 hours. For chemical stimulation 50 mM NH_4_Cl, KH_2_PO_4_, NH_4_NO_3_, NaCl, KCl and distilled H_2_O were used. Potassium, nitrogen and phosphorus co-limit the growth of carnivorous plants in natural habitat and carnivorous plants can obtain significant amount of K, N and P from insect prey [27], [28]. The concentrations were chosen on the basis of Lichtner and Williams experiments who found that 50 mM concentration was the most effective in induction of trap narrowing [17]. A soaked strip of filter paper was placed on the trap surface inducing the trap to shut. The opposite side of the paper strip was submerged into a 2 mL Eppendorf tube containing particular salts. After 24 hours, the strip of filter paper was removed and the percentage of traps narrowed in response to different treatments was evaluated. For induction of trap narrowing by jasmonic acid (JA) (Sigma-Aldrich), 5 - 6 drops of 2 mM (±)-JA containing 0.001% Tween were applied on the trap surface. The pure 0.001% Tween had no effect on trap narrowing. The digestive fluid was collected 24 and 48 hours after mechanical and chemical treatments and 48 hours after JA-induced narrowed traps using a pipette forced in between the trap lobes for further analysis. It was found that the rate of secretion is the highest between 24 – 96 hours [20].

### Measurements of electrical activity

The APs were recorded by a non-invasive device inside a Faraday cage. The APs were measured on the trap surface with non-polarizable Ag–AgCl surface electrodes (Scanlab systems, Prague, Czech Republic) fixed with a plastic clip and moistened with a drop of conductive EV gel (VUP, Prievidza, Slovakia) that is commonly used in electrocardiography. The reference electrode was taped to the side of the plastic pot containing the plant submerged in 1–2 cm of water in dish beneath the pot. The electrodes were connected to an amplifier made in-house (gain 1–1000, noise 2–3 µV, bandwidth [-3 dB] 10^5^ Hz, response time 10 µs, input impedance 10^12^ Ω). The signals from the amplifier were transferred to an analog-digital PC data converter (eight analog inputs, 12-bit-converter, ±10 V, PCA-7228AL, supplied by TEDIA, Plzeň, Czech Republic), collected every 6 ms. The sensitivity of the device was 13 µV. Moistened electrodes were equilibrated on the measured leaves for about an hour before measurement [29]. The mechanical stimulation was done by touching the trigger hairs with a pipette tip protruding outside the closed trap. The chemical stimulation was done by placing the soaked strip of filter paper inside the open trap, which subsequently snaps shut. Long-term observations (∼ 50 hours) were made on the potential changes that occur during chemical stimulation. All measurements were done in closed traps.

### Quantification of phytohormones

From 9 to 10 traps of given variant (including control open traps without any stimulation) were collected for hormone analysis in two independent experiments between 36 and 48 hours. Because it is known that wounding plant tissue caused by leaf cuts can increase the level of jasmonates, we used a fast method of sample collection [30]. The samples were cut and immediately (up to 10 seconds) frozen in liquid nitrogen and stored at -80 °C until extractions. Frozen plant material (15–20mg) was homogenized using an MM 301 vibration mill (Retsch GmbH & Co. KG, Haan, Germany) at a frequency of 27 Hz for 3 min and extracted with 1 ml of ice cold 10% MeOH/H_2_O (v/v) extraction solution in the presence of stable isotopically labelled internal standards ([^2^H_6_]-(±)-JA, [^2^H_2_]-(-)-JA-Ile, [^2^H_5_]-IAA, [^2^H_4_]-SA, [^2^H_6_]-(+)-*cis*,*trans*-ABA), provided by Olchemim (Olomouc, Czech Republic). The samples were incubated 20 min at 4 °C by shaking using a laboratory rotator and centrifuged (3 min, 20000 rpm, 4 °C). Collected supernatants were further purified by solid-phase extraction (SPE). After sample application onto equilibrated 30 mg polymer-based solid-phase sorbent (Oasis HLB, Waters), columns were washed with 1 ml of extraction solution and analytes were eluted by 3 ml of 80% MeOH/H_2_O (v/v). Samples were evaporated up to dryness under the stream of nitrogen and dissolved in 30 µl of mobile phase (15% acetonitrile: 85% 10 mM HCOOH, v/v). Final analysis of selected phytohormones was performed by an Acquity UPLC System (Waters, Milford, MA, USA) coupled to quadrupole mass spectrometer Xevo TQ MS (Waters MS Technologies, Manchester, UK) in the following conditions. Reversed-phase separation was performed using Acquity UPLC CSH C_18_ (100 x 2.1 mm; 1.7 µm; Waters) column and analytes were eluted with linear (0 to 5 min, 15% A; 5 to 15 min; 45% A), logarithmic (15 to 28 min, 48.6% A) and linear (28 to 29 min; 100% A) gradient of acetonitrile (A) and 10 mM formic acid (B), as mobile phases, at a flow rate 0.4 ml min^-1^. Phytohormones were determined in multiple reaction monitoring mode using mass transitions: 215.2 > 58.8, 326.2 > 151.1, 181.2 > 134.1, 141.1 > 96.8, 269.2 > 159.1, 209.2 > 58.8, 324.3 > 151.2, 293.3 > 275.3, 176.3 > 130.2, 137.1 > 92.8, 263.2 > 153.1 for [^2^H_6_]-(±)-JA, [^2^H_2_]-(-)-JA-Ile, [^2^H_5_]-IAA, [^2^H_4_]-SA, [^2^H_6_]-(+)-*cis*,*trans*-ABA, (-)-JA, (-)-JA-Ile, *cis*-(+)-OPDA, IAA, SA and ABA, respectively. Optimized parameters of MS/MS measurements were following: capillary/cone voltage, 3 kV/23–30 V; source/desolvation temperature, 120/550 °C; cone/desolvation gas flow, 70/650 L h^-1^; collision energy, 12–23 eV; collision gas flow, 0.21 mL min^-1^. Quantification was carried out by using isotope dilution method and a calibration curves with a good correlation coefficients (0.9963–0.9999) were obtained. Three independent technical measurements were performed for at least nine biological replicates from two independent experiments.

### Enzyme assay

To determine enzymatic activity of secreted digestive fluid in response to mechanical, chemical and JA stimulation, a common spectrophotometric method was used. We used three chromogenic substrates, 4-nitrophenyl phosphate, 4-nitrophenyl β-D-glucopyranoside, 4-nitrophenyl N-acetyl-β-D-glucosaminide (Sigma-Aldrich, St. Louis, USA), to estimate the activity of acid phosphatases (APs), β-D-glucosidases (BGs), N-acetyl-β-D-glucosaminidases (NAGs), respectively. All substrates were prepared in 50 mM (pH  =  5.0) acetate buffer, and the concentration of each was 5, 5, 2 mM, respectively. Only fresh digestive fluids were used for measurements, because just one freeze-thaw cycle significantly decreased the phosphatase activity. Portion of 15 µL (for APs), and 200 µL (for BGs and NAGs) of collected fluid was added to 535 µL (for APs), and 350 µL (for BGs and NAGs) of 50 mM acetate buffer (pH  =  5.0), and mixed with µL of a particular substrate. For controls, 400 µL of each substrate solution was mixed with 550 µL of the buffer. Mixed samples were incubated at 25 °C for 15 min (for APs) or 4 hours (for BGs and NAGs), and then 160 µL of 1.0 N NaOH was added to terminate the reaction. Absorbance was measured at 405 nm with spectrophotometer Jenway 6705 UV/Vis (Bibby Scientific Ltd, Essex, UK). A calibration curve was determined using 4-nitrophenol.

Because BG and NAG activity was not detected spectrophotometrically, we also used a more sensitive fluorometric method. Fluorogenic substrates 4-methylumbelliferyl N-acetyl-β-D-glucosaminide and 4-methylumbelliferyl β-D-glucoside were used to estimate the activity of N-acetyl-β-D-glucosaminidases and β-D-glucosidases, respectively. We used black 96-well microplates for fluorescence detection (Nunc, Roskilde, Denmark). The wells were filled with 300 µL of 50 mM acetate buffer (pH  =  5). Then 10 µL of digestive fluid was added and the wells were supplemented with one of the two above substrates at 50 µL each (300 µM final concentration) and mixed afterwards. Distilled water at 50 µL each was added to control wells to measure background fluorescence. Immediately after adding all substrates, the microplate was inserted into the microplate reader to detect the rate of hydrolysis of each substrate during the next 2 hours. Relative fluorescence in each well was measured (excitation/emission wavelengths were 365 nm and 450 nm) at 5-min intervals with fluorescence microplate reader Infinite F200 (Tecan Group Ltd., Männedorf, Switzerland). A linear increase in fluorescence over time indicated hydrolysis of the substrate. For calibration of the method, a microplate was filled with appropriate amounts of sample and buffer but, instead of the corresponding substrate, 50 µL of 4-methylumbelliferone was used as the standard. We determined the calibration factor as a slope of the regression line (R^2^  =  0.99).

Proteolytic activity of leaf exudates was determined by incubating 150 µL of a sample (75 µL of secreted fluid + 75 µL of 50 mM sodium acetate buffer, pH  =  5.0) with 150 µL of 2% (w/v) bovine serum albumin (BSA) in 200 mM glycine-HCl (pH  =  3.0) at 37 °C for 1 hour. The reaction was stopped by the addition of 450 µL of 5% (w/v) trichloroacetic acid. Samples were incubated on ice for 10 min, and centrifuged at 20. 000 g for 10 min at 4 °C. Absorbance of the supernatant at 280 nm was measured by spectrophotometer Jenway 6705 UV/Vis (Bibby Scientific Ltd, Essex, UK). For inhibitory studies, 3 µL of 1 mM cysteine peptidase inhibitor E-64 (Sigma-Aldrich, St. Louis, MO, USA) was added to 297 µL [75 µL of secreted fluid + 72 µL of 50 mM sodium acetate buffer, pH  =  5.0 with 150 µL of 2% (w/v) bovine serum albumin (BSA) in 200 mM glycine-HCl (pH  =  3.0)] to have a final effective 10 µM inhibition concentration [24]. One unit of proteolytic activity is defined as an increase of 0.001 per min in the absorbance at 280 nm [31].

Total protein content in digestive fluid was determined using Bicinchoninic Acid Kit for Protein Determination (Sigma-Aldrich, St Louis, MO, USA) according to the instruction manual and absorbance was measured at 562 nm by spectrophotometer Jenway 6705 UV/Vis (Bibby Scientific Ltd, Essex, UK). The activities were recalculated into µmol mg^-1^ proteins h^-1^ or U mg^-1^ proteins for phosphatase and proteolytic activity, respectively. All the experiments were repeated five times from independently collected fresh digestive fluid.

### SDS-PAGE and Western blots

For detection and quantification of cysteine endopeptidase called dionain-1 [24], [25], the polyclonal antibody against this protein was raised in rabbits by Agrisera (Vännäs, Sweden). The following aminoacid sequence (epitope) was synthesized: (NH_2_-) CAFQYVVNNQGIDTE (-CONH_2_), coupled to a carrier protein (keyhole limpet hemocyanin, KLH) and injected into two rabbits. The terminal cysteine was used for conjugation. The serum was checked for the presence of antigen specific antibodies using the ELISA test.

The digestive fluid was denatured by adding the loading buffer and heated at 70 °C for 30 min. The same amount of protein (quantified by Bicinchoninic Acid Kit) was electrophoresed in 12% (v/v) SDS-polyacrylamide gel followed by transfer to the Hybond-C membranes (Amersham, Freiburg, Germany) by Trans-Blot SD Semi-Dry Electrophoretic Transfer Cell (Biorad, Hercules, CA, USA). Because protein concentration in collected samples of digestive fluid was very low, below detectable level of Coomassie staining, the gels were visualized by silver staining (Proteosilver, Sigma Aldrich, St. Louis, MO, USA). For protein immunodetection, the polyclonal primary antibody against dionain-1 was used. After blocking in TBS-T containing 5% skim milk, the membrane was incubated with the primary antibody for 1 hour at room temperature. After washing, the membrane was incubated with the secondary antibody; the goat antirabbit IgG (H + L)-horseradish peroxidase conjugate (Bio-Rad, Hercules, CA, USA). Blots were visualized using Immobilon Western chemiluminiscent HRP substrate (Millipore, Billerica, MA, USA) and medical X-ray film (FOMA BIOCHEMIA, Hradec Králové, Czech Republic).

### Protease analysis by zymogram SDS-PAGE

Protease activities were determined after separation of 30 µL aliquots (digestive fluid sample mixed with zymogram sample buffer 1:1) in 12 % (v/v) SDS -polyacrylamide gel with casein as substrate in the separating gel. After separation, the proteins were renatured by placing the gels in 2.5 % Triton X-100 for 30 min at room temperature. The gels were incubating in development solution at 37°C, and then stained with Coomassie Brilliant Blue R-250 staining solution, and destained until the clear band appeared against blue background indicating protease activity. Ready gel zymograms and all kits and reagents were purchased from Biorad (Hercules, CA, USA).

### Statistical analysis

Throughout this paper, the mean with standard error (s.e.) is provided wherever possible. Enzymatic activities and hormone analysis among treatments were evaluated by one-way analysis of variance followed by Tukey-test (Origin 8.5.1., OriginLab corporation, Northampton, MA, USA). Comparison between mechanically and JA-induced traps was done using Student's t-test (Microsoft Excel). Prior to the statistical tests, the data were analysed for normality and homogeneity of variance. When a non-homogeneity was present, a *t*-test was employed with the appropriate corrected degrees of freedom. The statistical significance of the relationship between proteolytic activity and endogenous phytohormone level was evaluated by significance test for linear regression (Origin 8.5.1., OriginLab corporation, Northampton, MA, USA).

## Results

### Factors affecting trap narrowing and releasing of digestive fluid

**Figure 1:**
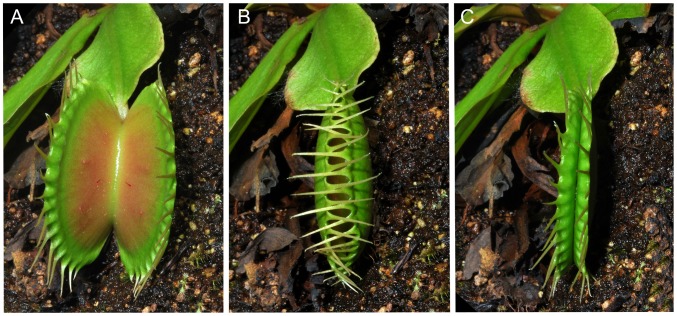
Traps of *Dionaea muscipula* in response to mechanical stimulation. An open trap (A), the same trap 1 min after double mechanical stimulation of trigger hairs (B) and 3 hours later after repeated mechanical stimulation in narrowed phase (C).

**Figure 2:**
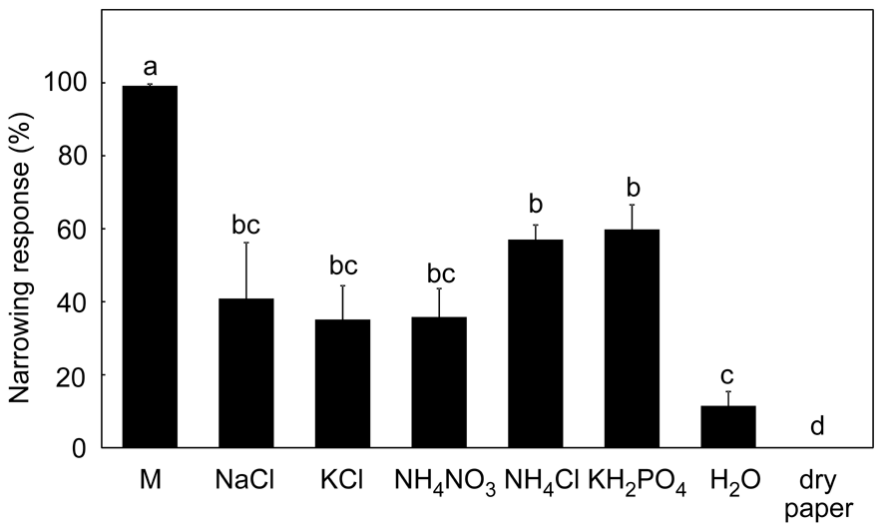
Narrowing response after different treatments. As a control wet (distilled H_2_O) and dry filter papers were applied. M – mechanical stimulation, Different letters denote significant differences at *P* < 0.05 (ANOVA, Tukey-test), means ± s.e., n  =  5–9.

Mechanical stimulation was very effective in induction of the narrowing phase (Fig. 1A,B,C), with 99.2 ± 0.4 % of traps induced. In comparison, the chemical stimuli induced from 37.0 to 60 % of traps. Mechanical stimulation also induce trap narrowing much faster, usually within 3 hours. Both type of stimulation induced secretion of digestive fluid. If the closed traps were without any additional mechanical or chemical stimulation (dry filter paper placed inside trap), the traps re-open within 24 hours without secretion of digestive fluid (Fig. 2). For futher analyses, we chose two most effective substances: KH_2_PO_4_ and NH_4_Cl (further P(K) and N(Cl) stimulation, respectively).

### Electrical activity

**Figure 3:**
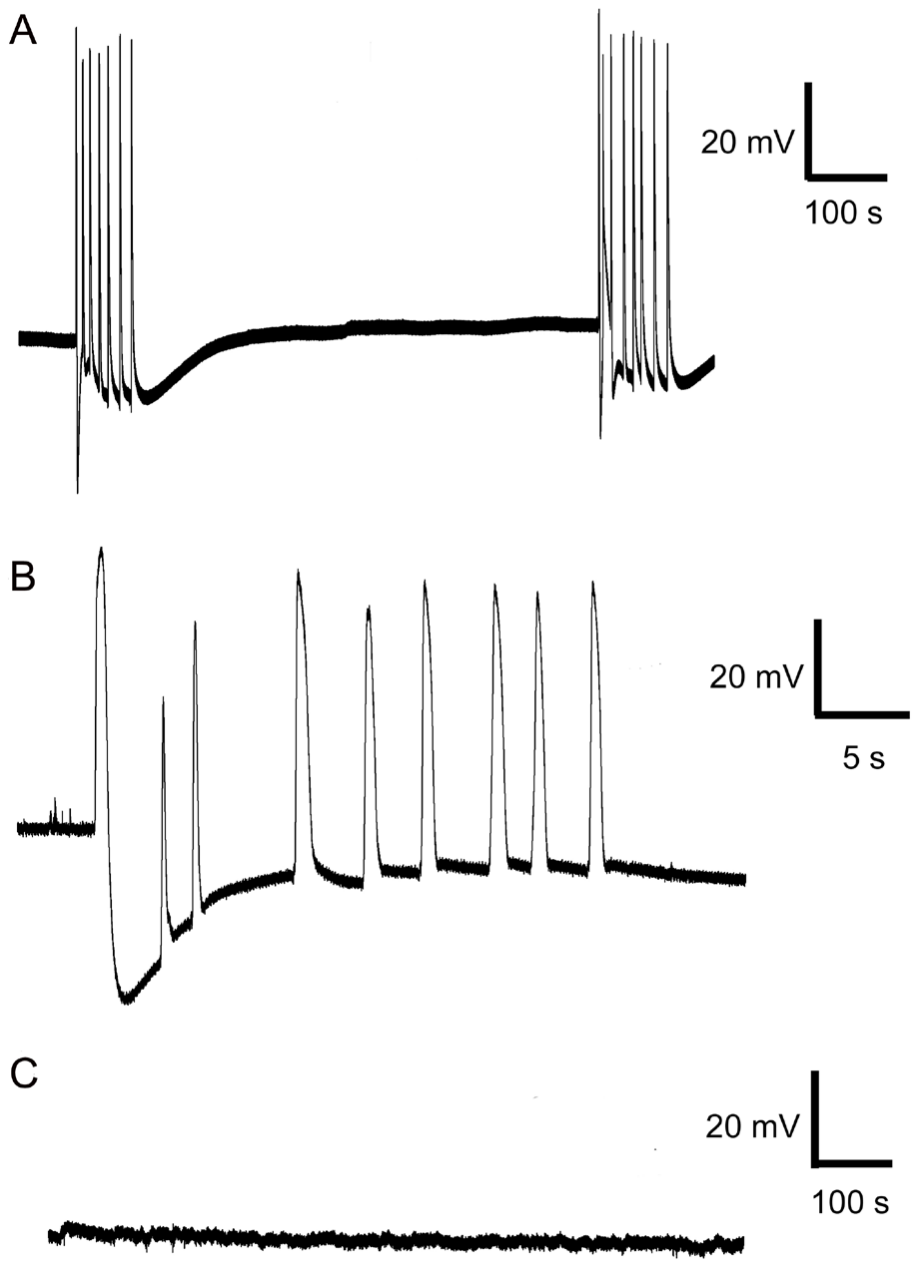
Electrical activity measured by extracellular electrode on trap surface. Mechanical irritation delivered in 10 minutes intervals (A), detailed view on action potentials (B) and chemical stimulation with NH_4_Cl and KH_2_PO_4_ did not trigger any AP (C).

Periodic mechanical stimulation delivered at 10–15 min intervals induced a series of APs (Fig. 3A,B). Chemical stimulation by KH_2_PO_4_ and NH_4_Cl did not induce any spontaneous APs (observation for 50 hours, 10 different traps, Fig. 3C).

### Induction of enzymatic activities

**Figure 4:**
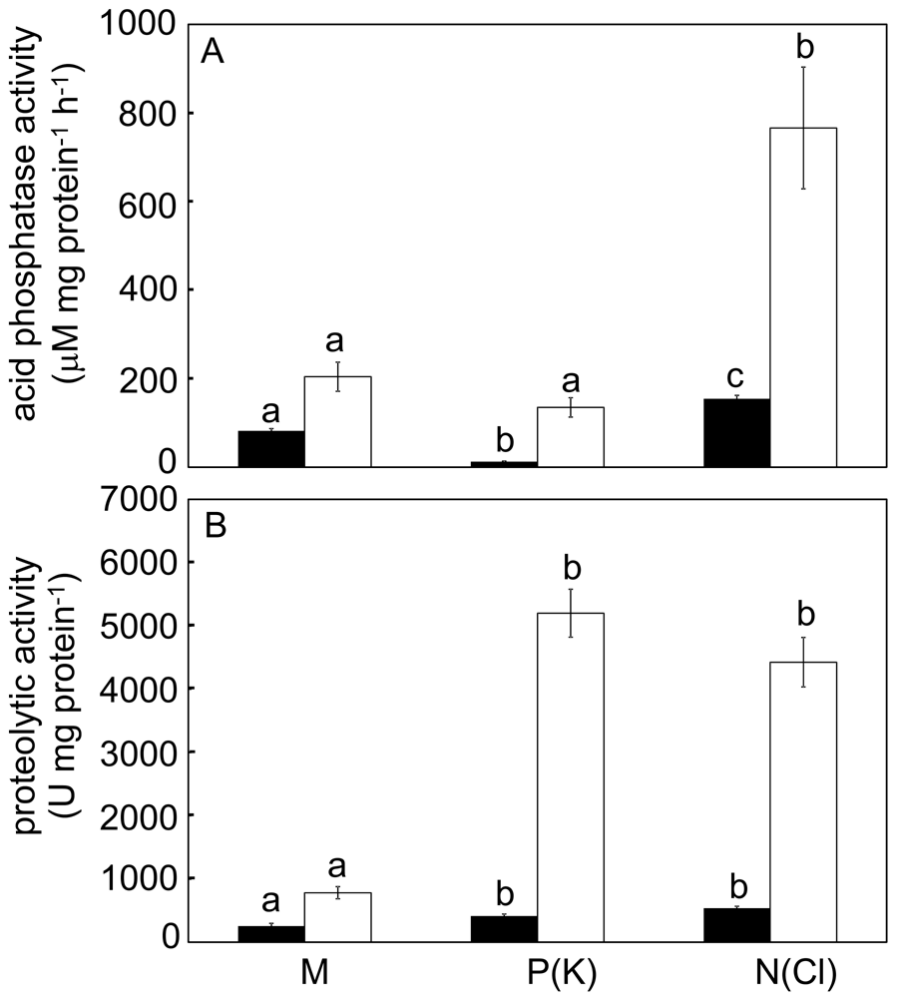
Enzymatic activities in response to mechanical and chemical stimulation after 24 (black bars) and 48 hours (white bars). Acid phosphatase activity (A) and proteolytic activity (B). Different letters denote significant differences at the same time interval at *P* < 0.05 (ANOVA, Tukey-test), means ± s.e., n  =  7–9.

**Figure 5:**
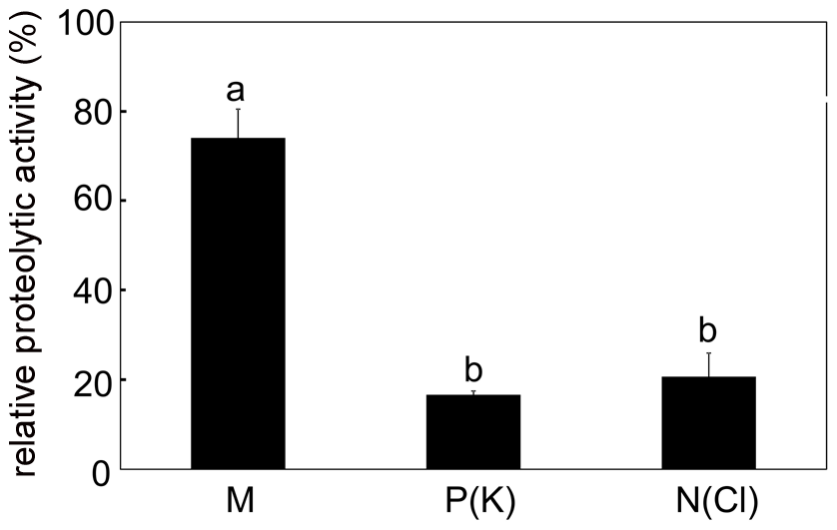
Inhibition of cysteine peptidase activity after 48 hours. Relative proteolytic activity measured after inhibition with 10 µM cysteine proteinase inhibitor E-64. Activity without inhibitor of given variant is 100 %. Different letters denote significant differences at P < 0.05 (ANOVA, Tukey-test), means ± s.e., n  =  6.

In general, higher enzymatic activities were recorded after 48 hours in comparison to 24 hours after induction. Both mechanical and chemical stimulation induced acid phosphatase activity. The highest activity was found after N(Cl)-stimulation followed by mechanical and P(K)-stimulation (Fig. 4A). The total proteolytic activity in mechanically induced traps was several times lower in comparison to chemical stimulation (Fig. 4B). The relative differences between treatments remained the same regardless the activity expressed per mg proteins or per ml (not shown). The proteolytic activity was inhibited with 10 µM cysteine peptidase inhibitor E-64 by 26% (mechanical stimulation) followed by 79% (N(Cl)-stimulation) and 83% (P(K)-stimulation), Fig. 5. β-D-glucosidase and N-acetyl-β-D-glucosaminidase (exochitinase) activities were not detected regardless of type of stimulation and method used for detection (spectrophotometric or spectrofluorimetric). As a control we used water soaked papers which induced enzyme activities comparable to mechanical stimulation. Induction of proteolytic activity by KCl was comparable to P(K) and N(Cl) stimulation (Fig. S1).

### Protein pattern and abundance of cysteine endopeptidase dionain

**Figure 6:**
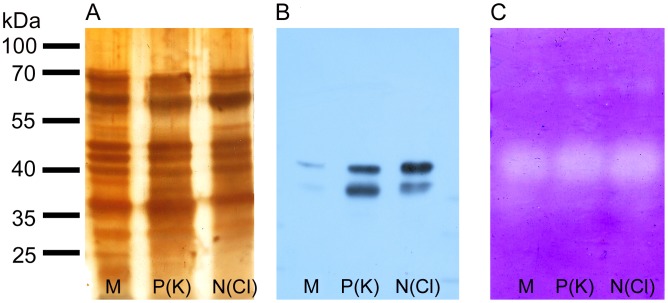
Protein pattern, Western blot and zymogram in response to mechanical and chemical stimulation. Silver-stained SDS-polyacrylamide gel containing proteins in *D. muscipula* digestive fluid after mechanical (M), P(K) and N(Cl) stimulation (A). The same amount of proteins was electrophoresed in 12% (v/v) SDS-polyacrylamide gel and subjected to Western blot analysis using antibodies against dionain-1 (B). Detection of protease activity in 12% (v/v) SDS-polyacrylamide gel with casein as a substrate (C). The clear bands against background indicate protease activity. Representative gels at least from 4 repetitions are shown.

There were no obvious qualitative differences in the protein profiles of digestive fluid released in response to mechanical or chemical stimulation (Fig. 6A). Although silver staining has a high sensitivity, it has also a narrow linear dynamic range, making it less suitable for protein band quantification. Therefore, the antibody against dionain-1 was raised for quantification of this crucial protein in two rabbits. We found two positive reactions around 40 kDa (Fig. 6B). The molecular weight 40 kDa is lower than found by Takahashi *et al*. (2011) [24] but agrees with that found by Scala *et al.* (1969) and Schulze *et al.* (2012) [20], [25]. We suggest that the lower band below 40 kDa may be due to degraded enzyme or enzyme with a lower degree of glycosylation (dionain contains several glycosylation sites). Another explantaion is that the antibody binds to the active and inactive forms of the peptidase, since the C1A enzymes including dionain have a propeptide that must be cleaved to obtain an active protein. Indeed, the dionain-1 was identified in three bands of lower molecular weight [25]. We cannot exclude the possibility that the antibody used binds to dionain-2, -3 or -4, which share 50% identity of our epitope against dionain-1 (the sequence of dionain 2, 3, 4 was not known at the time of our antibody production; published by Schulze *et al.*, 2012 [25]). The abundance of dionain is obviously much higher in response to chemical than mechanical stimulation, that is in agreement with significantly higher proteolytic activity in digestive fluid released in response to chemical stimulation (Fig. 4B). The position of dionain approximately agrees with the position of clear bands on zymogram which is caused by protease mediated casein degradation. The clear bands around 70 kDa belong probably to serine carboxypeptidase-like 49 protein as was identified by Schulze *et al*. (2012) [25], and its activity is also higher after chemical in comparison to mechanical stimulation (Fig. 6C).

### Jasmonates accumulate in response to mechanical and chemical stimuli

**Figure 7:**
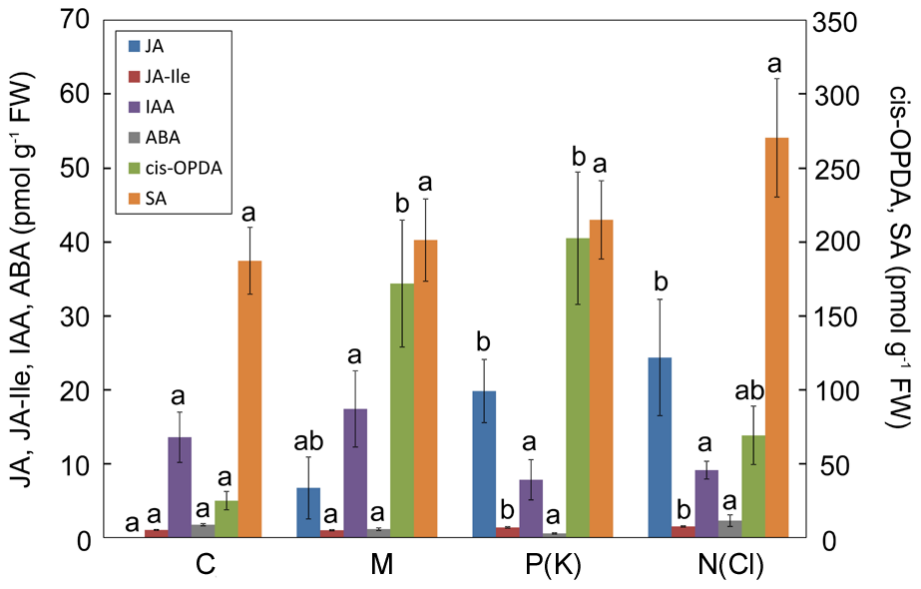
Endogenous concentration of phytohormones (JA, JA-Ile, IAA, ABA, *cis*-OPDA and SA) in trap tissue 36 - 48 hours after stimulation. Note different scale on y-axis for *cis*-OPDA and SA. Control (C), mechanical (M), P(K) and N(Cl) stimulation. Different letters denote significant differences at *P* < 0.05 of particular phytohormone among treatments (ANOVA, Tukey test), means ± s.e., n  =  9–10.

**Figure 8:**
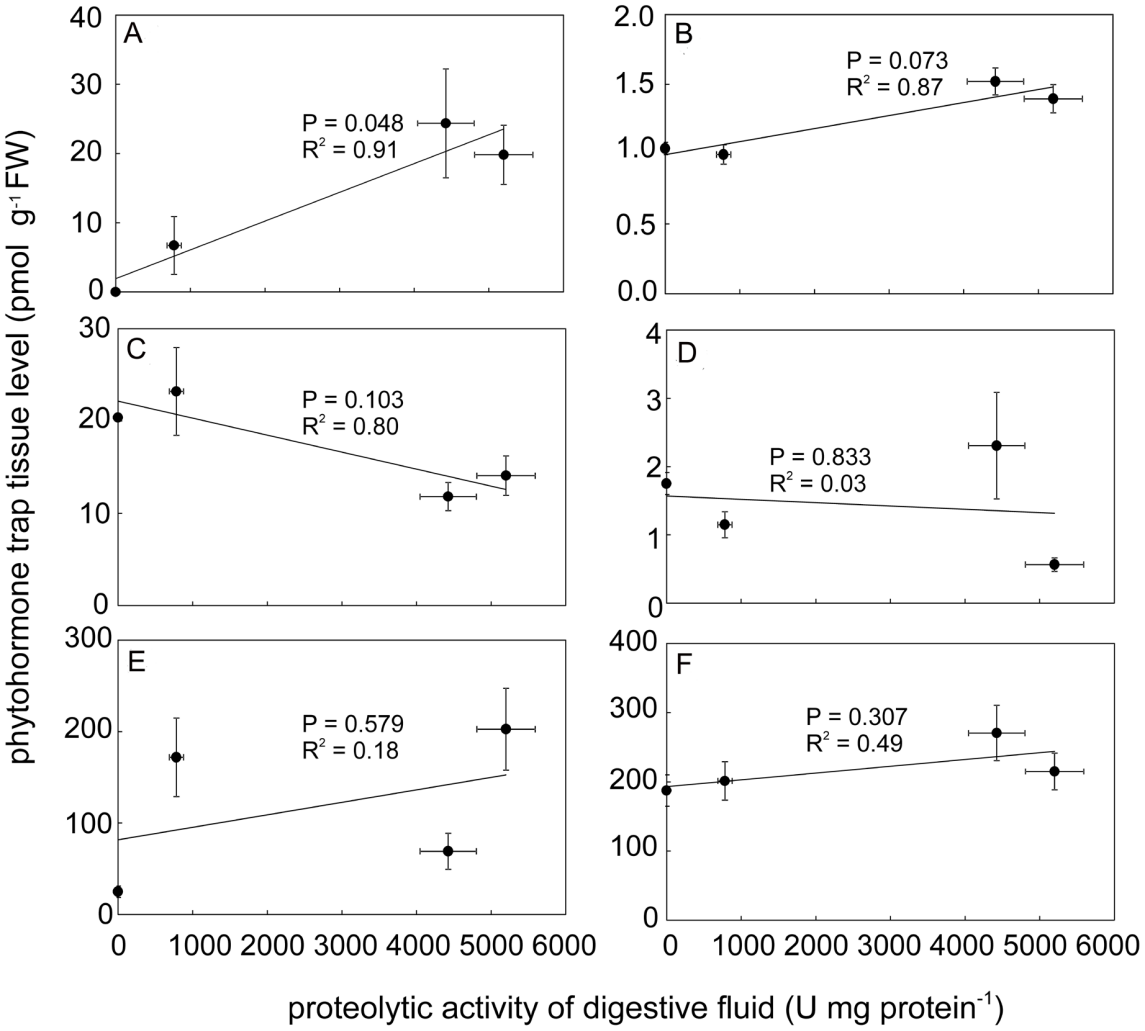
Correlation between endogenous phytohormone level and proteolytic activity. Correlation between endogenous concentration of JA (A), JA-Ile (B), IAA (C), ABA (D), *cis*-OPDA (E), SA (F) and proteolytic activity induced by mechanical and chemical stimulation. The linear relationship is significant only for JA (*P*  =  0.048, significance test for linear regression), means ± s.e.

The fact that jasmonic acid (JA) was not detected in any resting control trap tissue indicates that our sampling method was sufficiently fast [30]. Analysis of treated traps showed accumulation of JA in response to mechanical stimulation and especially to N(Cl) and P(K) stimulation. For isoleucine conjugate of jasmonic acid (JA-Ile) a significant but only very small increase (app. 50 %) was measured in N(Cl) and P(K) stimulated traps. The rise of *cis*-OPDA was also evident, especially in mechanically and P(K) induced traps. Taken into account the absolute concentration of all studied jasmonates in trap tissue, the *cis*-OPDA raised to the highest level, followed by JA and finally to very small level of JA-Ile. The accumulation of indole-3-acetic acid (IAA), salicylic acid (SA) and abscisic acid (ABA) did not change significantly in response to mechanical or chemical stimulation (Fig. 7). The linear regression analysis showed that the relationship between phytohormone concentration and the proteolytic activity is significant only for JA (Fig. 8).

### Jasmonic acid triggers secretion of cysteine endopeptidase dionain

**Figure 9:**
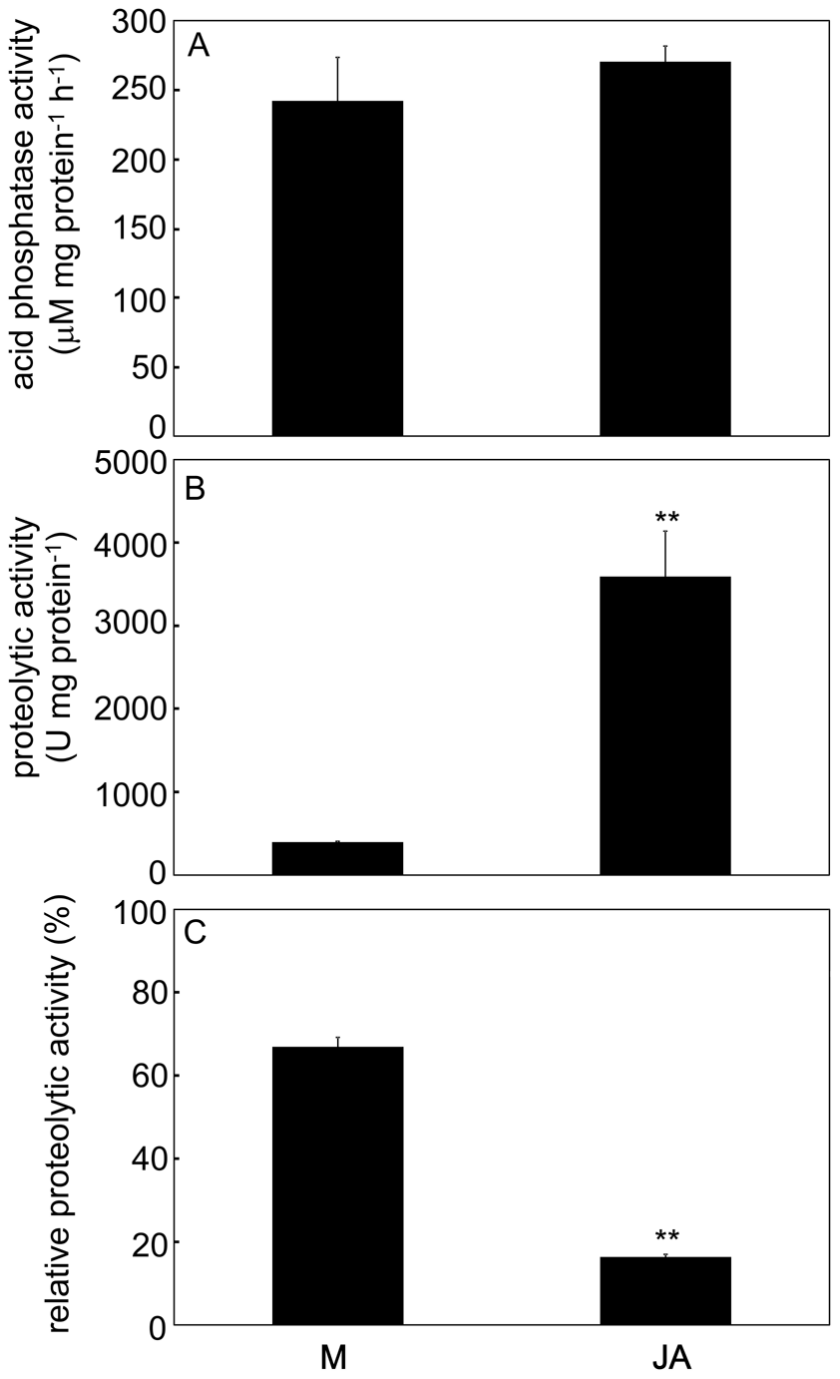
Enzymatic activities in response to external JA application. Acid phosphatase activities (A), proteolytic activities (B) and activities measured after inhibition with 10 µM cysteine proteinase inhibitor E-64. Activity without inhibitor of given variant is 100 % (C). ** - significant differences at *P*  =  0.01 (Student's t-test), means ± s.e., n  =  6–7.

**Figure 10:**
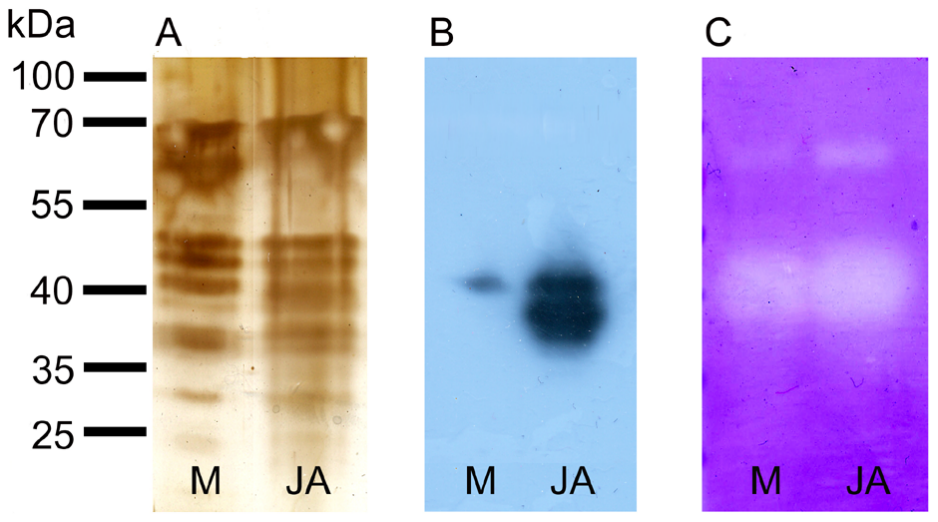
Protein pattern, Western blot and zymogram in response to external application of jasmonic acid. Silver-stained SDS-polyacrylamide gel containing proteins in *D. muscipula* digestive fluid after mechanical stimulation (M) and external application of jasmonic acid (JA), (A). The same amount of proteins was electrophoresed in 12% (v/v) SDS-polyacrylamide gel and subjected to Western blot analysis using antibodies against dionain-1 (B). Detection of protease activity in 12% (v/v) SDS-polyacrylamide gel with casein as a substrate (C). The clear bands against background indicate protease activity. Representative gels at least from 4 repetitions are shown.

Application of (±)-JA on the trap surface induced trap closing within 12 hours and led to the formation of narrowed phase with 100% of traps induced. The digestive fluid released 48 hours after external application of JA significantly upregulated the proteolytic activity but not phosphatase activity (Fig. 9A,B). The proteolytic activity was supressed by application of 10 µM cysteine peptidase inhibitor E-64 (Fig. 9C). The Western blots confirmed a higher abundance of dionain in JA-treated plants in comparison to mechanical stimulation (Fig. 10A,B). This again agrees with the position of clear bands on the zymogram. The proteolytic activity around 70 kDa belonging probably to serine carboxypeptidase-like 49 protein was also upregulated in JA-treated traps (Fig. 10C).

## Discussion

The trapping mechanism and electrical signalling in the Venus flytrap have intrigued scientist since they were first described by Burdon-Sanderson (1873) and Charles Darwin (1875) [1], [2]. The prey capture and digestion are usually mediated by two types of stimuli: mechanical and chemical. In this study we distinguish between these two stimuli to find out their regulatory role in enzyme secretion. The mechanical stimulation of trigger hairs is important mainly in the first hours after prey capture and is responsible for fast trap narrowing and releasing of the first digestive fluid (Fig. 1). This type of stimulation is coupled with the generation of APs (Fig. 3A,B) and increased cytosolic Ca^2+^ level [3], [19]. Although Juniper *et al*. (1989) [32] suggested that there is no enzyme release following mechanical stimulation, our study and that of Schulze *et al*. (2012) found the enzymes in digestive fluid [25]. Once the entrapped prey died, chemical stimuli released from prey decomposition keep the trap in narrowed phase with enzyme secretion [17]. Volkov *et al*. (2011) also noted rapid induction of narrowed phase after application of external charge [33]. No APs have been recorded in response to 50 mM NH_4_Cl and KH_2_PO_4_ in our study (Fig. 3C). Although Balotin and DiPalma (1962) have demonstrated production of spontaneous APs in response to chemical stimulation (510 mM NaCl), these results are more likely due to osmotic shock rather than to chemical stimulation [17], [34]. Affolter and Olivo (1975) have clearly demonstrated that no APs were detectable on the trap surface after the death of an insect which previously stimulated and triggered more than one hundred APs [15]. However, recently, the depolarization of membrane potential and even AP in 2-days prey-activated digestive glands in response to 10 mM NH_4_Cl was documented by current-clamp microelectrode caused by the activity of DmAMT1 ammonium channel [35].

Mechanical stimulation with subsequent electrical activity triggers secretion of digestive fluid but is not sufficient to trigger full proteolytic capacity in *D. muscipula* (Fig. 4B). Robins (1976) showed differences between quantities of fluid and proteins produced in response to different nitrogenous stimuli in *D. muscipula*, indicating that fluid and enzyme release are not under the same control [16]. It is tempting to assume that the APs trigger basal secretion of acid phospatases and proteolytic enzymes, which are later under the control of chemical stimuli released from prey. The range of chemical stimuli is wide and include different salts and nitrogenous compounds. Darwin tested 51 salts and found that 25 were effective in induction of tentacle movement in carnivorous sundew *Drosera rotundifolia* [1]. In the genus *Drosera* proteolytic, chitinase, phosphatase and phosphodiesterase activities were not or only slightly increased in response to mechanical stirring of sticky tentacles. However, application of chemical stimuli (live prey, gelatine or chitin) significantly increased the enzymatic activities [28], [31]. Acid phosphatase activity was not much induced by P(K)-stimulation in our study (Fig. 4A), that is probably caused by the fact that P is a known competitive inhibitor of acid phosphatases [36]. No induction of enzyme secretion was observed after mechanical stimulation of passive pitcher trap in *Sarracenia purpurea*. The passive traps are not able to generate APs in response to mechanical stimulation in contrast to *Drosera* or *Dionaea*, however chemical stimuli in the form of bovine serum albumin (BSA) or NH_4_^+^ significantly increased phosphatase and proteolytic activity [37]. Induction of endochitinase activity by chitin was recently described in carnivorous pitcher plants of the genus *Nepenthes* [38], [39], [40], [41]. In accordance with our study, exochitinase (or N-acetyl-β-D-glucosaminidase) activity in carnivorous plants has not been detected in *Dionaea*, *Drosera* and *Nepenthes* [21], [28], [42]. Robins and Juniper (1980) showed using autoradiography for the first time that proteins released from the glands comes from a store but considerable *de novo* synthesis must also occur during the digestion period [43], [44]. This was recently confirmed by the presence of mRNA for dionain and VF chitinase I in stimulated trap tissue [25], [42]. Nishimura *et al*. (2013) found induced transcription of S-like RNase genes in response to application of BSA in Venus flytrap [45]. These studies have shown that enzyme secretion in carnivorous plants is precisely regulated in response to stimuli from prey at the level of transcription.

Aspartic proteinases have been involved in protein digestion in *Nepenthes*, *Cephalotus*, *Drosera* and *Dionaea* [22], [23], [25]. Stephenson and Hogan [46] were the first to suggest that cysteine proteinase is involved in *Nepenthes ventricosa*. However cysteine proteinase has not been found in digestive fluid of *Nepenthes* analysed by mass spectrometry [41], [47] on the contrary to the digestive fluid of *D. muscipula* [22], [25], suggesting that if cysteine endopeptidase occurs in digestive fluid of *Nepenthes*, it plays only the minor role in protein digestion. Schulze *et al*. (2012) concluded that the aspartic proteinase dionaeasin play only a minor role in protein degradation in *D. muscipula* [25]. The dominant role of cysteine endopeptidase in protein digestion in Venus flytrap is confirmed also by our inhibitory experiments (Fig. 5). Our western blot analysis showed its higher accumulation in response to chemical in comparison to mechanical stimulation (Fig. 6B).

The link between electrical signalling and jasmonate accumulation is now well recognized [29], [48], [49], [50]. Jasmonates are lipid-derived compounds acting as key signaling molecules in plant stress responses [51]. Recently, it has been shown that they play also an indispensable role in leaf bending and digestive fluid secretion in carnivorous plants [19], [42], [52], [53]. In this paper, the term jasmonates include not only JA and JA-Ile but also oxylipin *cis*-OPDA. Escalante-Pérez *et al*. (2011) found accumulation of OPDA already after 30 min from prey capture in Venus flytrap and its level remained high until one week [19]. *Cis*-OPDA accumulated also in our study in both type of stimulation (Fig. 7). Although they did not show their JA and JA-Ile data in response to prey capture, we found higher accumulation of JA in chemically induced traps in comparison to control resting traps (Fig. 7). Although we found an approximately 50% increase of JA-Ile, its concentration remained very low. This questioned its role in induction of enzyme secretion, but coronatine, structural mimic of JA-Ile (and not OPDA as was previously thought), induced secretion of digestive fluid and transcription of VF chitinase I in Venus flytrap [19], [42]. It is now generally accepted that JA-Ile instead of JA is ligand to the JA receptor. The perception of JA-Ile is achieved by the ubiquitin ligase SCF^COI1^ complex. When the F-box protein Coronatine-Insensitive1 (COI1) recognizes JA-Ile, it triggers the ubiquitination and subsequent proteosomal degradation of Jasmonate Zim Domain proteins (JAZ). The degradation of JAZ proteins relieves JAZ mediated repression of gene expression, leading to the activation of JA response. Neither JA nor OPDA are the ligand for COI1-JAZ co-receptor [54], [55], [56], [57]. However, the hypothesis that JA is bioactive *per se* cannot be completely rule out, as indicate studies of Chung *et al.* (2008) and Wang *et al*. (2008) [58], [59]. Moreover, it has been shown that OPDA elicits the transcript levels of many JA-responsive and JA-nonresponsive genes in *Arabidopsis* in a COI1-JAZ dependent or COI1-JAZ independent manner [60], [61]. A distinct set of genes is expressed by OPDA, but a partial overlap appeared with the expression of JA-induced genes in *Arabidopsis thaliana*. Transcript level of intracellular cysteine endopeptidase in *A. thaliana* from the same C1A enzyme subfamily as dionain increased 2.5 and 4.9 times in response to external application of OPDA and JA, respectively (supplemental data in [61]). The same holds for serine carboxypeptidase-like 49 protein from S10 family identified in digestive fluid of *Dionaea* [25]. The transcript level of serine carboxypeptidase-like 9 protein from the same S10 enzyme family in *A. thaliana* increased 2.3 and 3.1 in response to external application of OPDA and JA, respectively (supplemental data in [61]). In our study *cis*-OPDA accumulated to much higher level than JA and JA-Ile (e.g. for P(K)-stimulated traps *cis*-OPDA level is tenfold higher in comparison to JA, and 200-fold higher in comparison to JA-Ile, Fig. 7, 8), but the fact that high accumulation of *cis*-OPDA in mechanically induced traps did not trigger the full proteolytic activity indicates also the participation of JA and (or) JA-Ile in chemical induction. This suggestion is supported by Nakamura *et al*. (2013) and Mithöfer *et al*. (2014) who found increased accumulation of endogenous JA and JA-Ile, leaf bending and formation of ‘outer stomach’ in response to chemical stimulation of live and edible prey in sundew *Drosera capensis* and supposed that JA and JA-Ile might be involved in chemonastic reactions rather than in thigmonastic movements [52], [53]. Our unpublished data showed that external application of (±)-JA on the leaves of *D. capensis* also induced high proteolytic activity.

Our results clearly showed that external application of JA upregulated the proteolytic activity and abundance of cysteine endopeptidase dionain in digestive fluid (Fig. 9, 10). We must also take into account that exogenous application of JA and the structural mimic of JA-Ile, coronatine, also induces the accumulation of OPDA [19], [62]. The fact that the level of endogenous jasmonates increased in response to prey capture and applied JA can bypass mechanical and chemical stimuli from insect prey to trigger full proteolytic activity, highlight the importance of jasmonate signalling in carnivorous plants digestion with active trapping mechanism. It is tempting to assume that the coordinated action of *cis*-OPDA, JA and JA-Ile fine-tune production of proteolytic enzymes. Multiple signal pathways may interact functionally with one another to optimize the Venus flytrap response to edible prey. It remains to be elucidated whether the jasmonates accumulate in whole trap tissue or are spatially restricted to digestive glands, where the expression of carnivory-related genes occurs.

**Figure 11:**
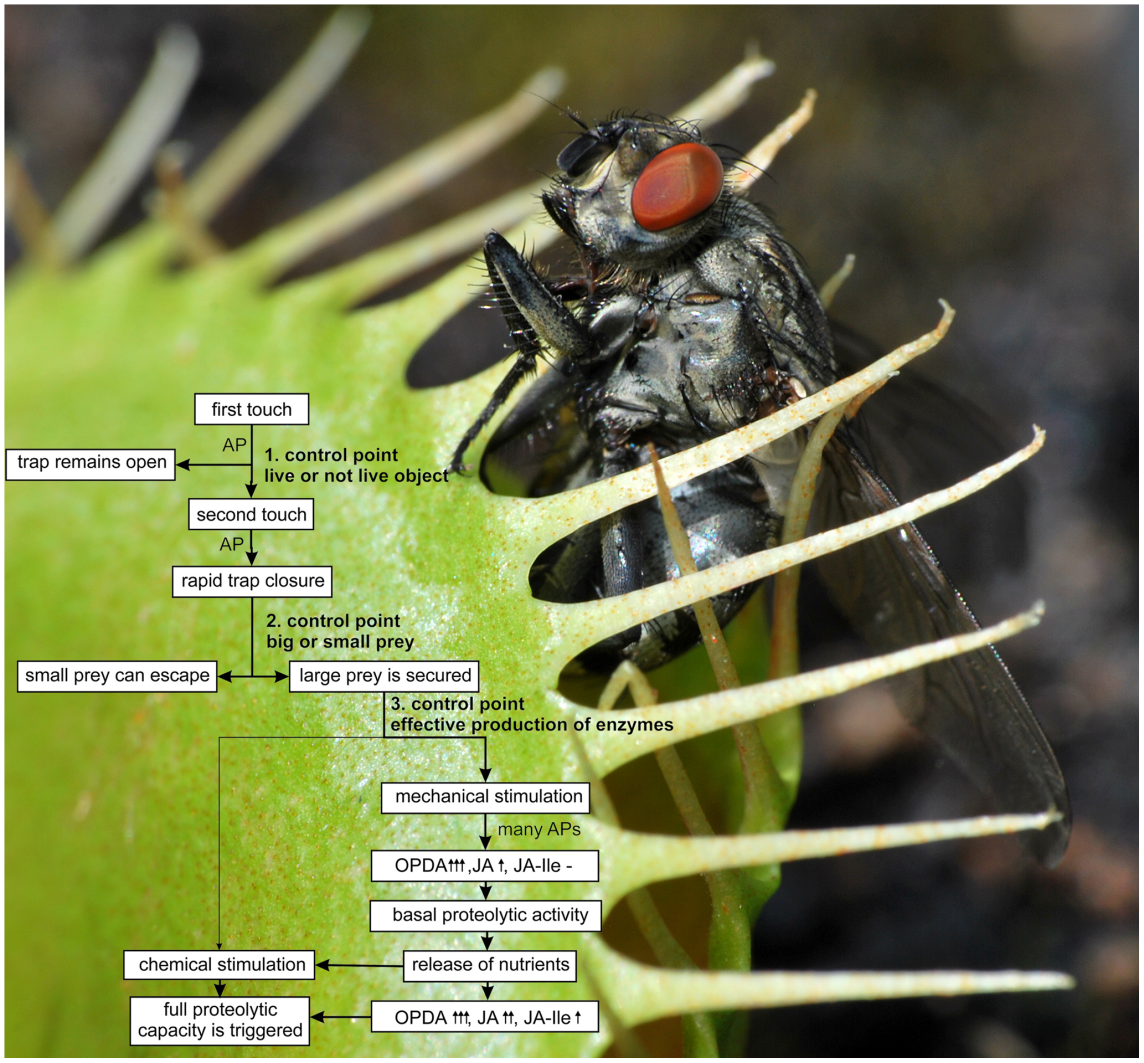
Double trigger mechanism for protein digestion. Three control points in prey capture and digestion of Venus flytrap, which ensure effective production of digestive enzymes. For detailed description, see discussion. AP – action potential, the increase of phytohormone level is indicated by arrows: no increase (-), small (↑), moderate (↑↑), high (↑↑↑).

The cost/benefit model proposed by Givnish *et al.* [63] became a framework for interpretation of many results in carnivorous plant research and the Venus flytrap is not an exception. Trap closure, prey retention and digestion in Venus flytrap are carbon costly. Trap closure and generation of APs transiently inhibit photosynthesis and stimulate mitochondrial respiration [64], [65]. It has been proposed that the stimulation of respiration is connected with rapid consumption of ATP during trap closure and AP generation [13], [64], [66]. The digestive fluid is also costly in term of carbon and nitrogen. On the other hand, the benefit in the form of increased uptake of N and P from prey can later stimulate photosynthesis, as has been documented in many carnivorous genera [28], [67], [68], [69], [70]. Thus, the Venus flytrap has evolved several control mechanisms how to optimize benefit from carnivory by reducing its associated costs, if there is nothing too much eat (Fig. 11). The first control point, double-trigger mechanism for prey capture in Venus flytrap, has been known for decades. At least two touches and two APs are necessary for trap closure at room temperature [4]. The lack of response to the first touch benefits the plants by preventing accidental closure through wind-blown sands, raindrops etc. [32]. If the trap snaps, the small gaps between marginal teeth of the trap allow small prey to escape from the trap [1], [71]. This second control point can help to save energy because small prey do not provide sufficient amounts of nutrients to repay the costs associated with prey retention and digestion and the trap slowly opens again [64]. However, if the bigger prey is secured, repeated mechanical stimulation of trigger hairs by struggling prey results in generation of hundreds APs and cause trap narrowing [15], [17], [19]. The mechanical stimulation is very effective in narrowing response and APs result in accumulation of jasmonates, mainly *cis*-OPDA, and trigger the releasing of digestive fluid. It has been suggested that the potential link between APs and jasmonate accumulation in plants is increased cytosolic Ca^2+^ level [48]. The digestive fluid released in response to mechanical stimulation has not the full power to digest protein. There must be some chemical or osmotically active substances released from partially digested or live prey, to trigger the full proteolytic activity – the double trigger mechanism in protein digestion and the third control point which ensure effective production of enzymes. The membrane depolarization and APs in prey-activated digestive glands may play a role in this process [35]. The gradual release of digestive enzymes with several control mechanisms (neuronal and hormonal) is also typical for animal digestive system [1]. It is tempting to assume that coordinated action of different jasmonates (JA, JA-Ile and *cis*-OPDA) regulate the transcription/exocytosis of cysteine endopeptidase dionain and optimize the digestive process in *D. muscipula*. In this way the Venus flytrap growing in nutrient poor habitats obtains up to 75% percent of leaf nitrogen from prey [72]. The surplus nitrogen can stimulate rate of photosynthesis and outweigh associated costs with prey retention and digestion [70].

## Supporting Information

Figure S1 Enzymatic activities in response to mechanical stimulation, papers soaked with water and KCl after 48 hours. Acid phosphatase activity (A) and proteolytic activity (B). Different letters denote significant differences at *P* < 0.05 (ANOVA, Tukey-test), means ± s.e., n  =  4.
(TIF)
