# Supplementary figures and images for: Abundance of Cysteine Endopeptidase Dionain in Digestive Fluid of Venus Flytrap (Dionaea muscipula Ellis) Is Regulated by Different Stimuli from Prey through Jasmonates
Source: PLoS One. 2014 Aug 25;9(8):e104424. doi: 10.1371/journal.pone.0104424 (PMC4143254; doi:10.1371/journal.pone.0104424)

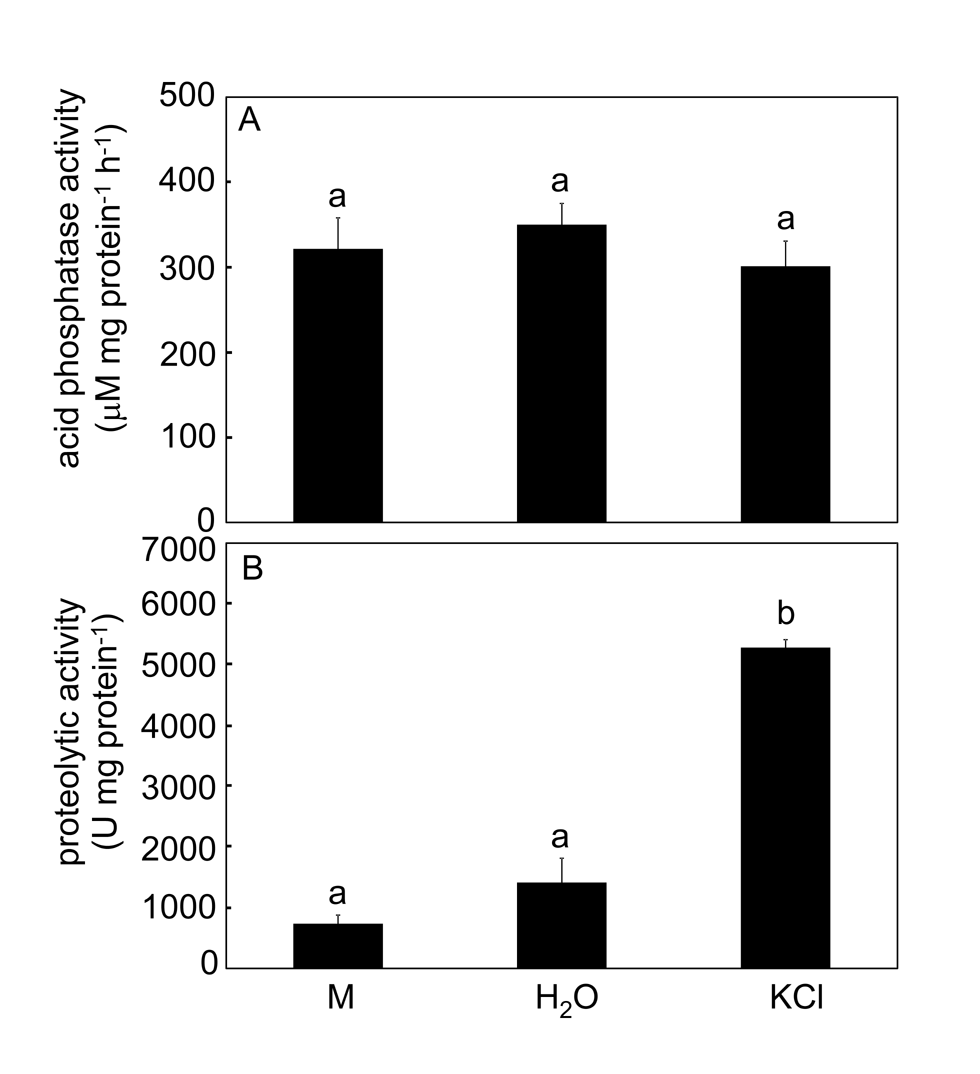

Supplement: Figure S1 — Enzymatic activities in response to mechanical stimulation, papers soaked with water and KCl after 48 hours. Acid phosphatase activity (A) and proteolytic activity (B). Different letters denote significant differences at P < 0.05 (ANOVA, Tukey-test), means ± s.e., n = 4. (TIF) [file pone.0104424.s001.tif]
